# Meta-Analysis of the Therapeutic Effect of Shenqi Jiangtang Granule on Type 2 Diabetes Mellitus

**DOI:** 10.1155/2020/5754823

**Published:** 2020-09-29

**Authors:** Ruo-Lan Li, Tai-Wei Dong, Ji-Gang Wei, Feng Gao, Min Li, Yang Bai, Pei-Feng Wei, Miao-Miao Xi

**Affiliations:** ^1^College of Pharmacy, Shaanxi University of Chinese Medicine, Shiji Road, Qindu District, Xianyang, Shaanxi 712046, China; ^2^Tongchuan Mineral Bureau Central Hospital, No. 15, Chuankou Road, Wangyi District, Tongchuan, Shaanxi 727000, China; ^3^The Second Affiliated Hospital of Shaanxi University of Traditional Chinese Medicine, Weiyang West Road, Qindu District, Xianyang, Shaanxi 712046, China

## Abstract

**Objective:**

To systematically evaluate the effectiveness of Shenqi Jiangtang granule (SQJT) in the treatment of type 2 diabetes.

**Methods:**

We searched CNKI, Wanfang Data, VIP, and PubMed databases to collect randomized controlled trials (RCT) of Shenqi Jiangtang granules in the treatment of type 2 diabetes. The search time was from January 2014 to the present. Data were extracted, and quality was evaluated. Metadata analysis of the extracted data was carried out using RevMa5.2 software. The final results are expressed in relative risk (RR), mean difference (MD), and 95% CI.

**Results:**

This study included a total of 13 studies, 1160 subjects. Meta-analysis results showed that the test group was better than the control group (RR = 1.26, 95% CI 1.18–1.34, *P* < 0.00001). The fasting blood glucose, postprandial blood glucose, and glycated hemoglobin of the test group were also significantly better than those of the control group.

**Conclusion:**

Shenqi Jiangtang granules have a certain clinical effect and low adverse reaction rate for the treatment or adjuvant treatment of type 2 diabetes. At present, the drug has been widely used in clinical practice, but a large number of large-sample clinical trials are needed to further verify its specific efficacy and safety.

## 1. Introduction

Diabetes is a metabolic disease characterized by high blood sugar, which is divided into type 1 and type 2. Type 1 diabetes is an autoimmune disease that causes destruction of islet *β* cells, accounting for about 5%–10% of patients with diabetes [1]. Type 2 diabetes mellitus (T2DM) is also known as adult-onset diabetes. It develops after 35 to 40 years of age and accounts for more than 90% of diabetic patients. This disease is characterized by hyperglycemia resulting from defects in insulin secretion, insulin action, or both [2]. The pathogenesis of T2DM is more complicated. With the development of social economy and the improvement of people's living standards, the prevalence of T2DM is rapidly increasing worldwide [3]. Therefore, we believe that the pathogenesis of the disease may have a close relationship with the patient's own lack of exercise, high-calorie diet, and poor eating habits. According to statistics from relevant data, the number of diabetes patients in 2107 has reached 42.49 million, and approximately 35.21 million people worldwide have impaired glucose tolerance (i.e., they are in prediabetes) [4]. It is emerging as one of the most prevalent human ailments next to cardiovascular diseases and is the sixth leading cause of death worldwide (WHO). It is estimated that, by 2030, the number of people with diabetes worldwide will be close to 550 million [5]. Diabetes is a chronic disease. Patients with long-term high blood sugar will cause more serious complications such as diabetic neuropathy, diabetic nephropathy, and diabetic foot [6, 7] which seriously threaten the patient's health and quality of life [8]. At present, the treatment of diabetes is mainly through long-term, regular use of drugs to improve the patient's glucose metabolism [9, 10]. The first-line drugs used in clinical treatment of T2DM mainly include acarbose, metformin hydrochloride, and sulfonylurea drugs. They have fast-acting effects and less side effects, but long-term use can also cause adverse reactions such as lactic acidosis, nausea, and bloating, not conducive to long-term treatment [11]. From the perspective of traditional Chinese medicine, diabetes is classified as “diabetes and dementia,” and its pathogenesis is attributed to Yin and Tianjin loss, hot and dry, qi deficiency, blood stasis, and blood stasis internal resistance [12–14]. The principle of treatment should be to invigorate qi, nourish yin, and nourish fluid to quench thirst [15]. The treatment of diabetes by traditional Chinese medicine focuses on the adjustment of the overall function, and the traditional Chinese medicine has a mild performance in reducing blood sugar and improving the metabolic function of the body, has a long-lasting effect and little adverse reactions, and can prevent and cure multiple organ complications [16], and we should carry out more in-depth clinical research. At present, in the treatment of T2DM in traditional Chinese medicine, Shenqi Jiangtang granules are widely used. Clinical trials on their efficacy have shown that Shenqi Jiangtang granules are superior to simple Western medicine treatments [17]. Considering a single study might have been underpowered to detect the overall effects, a quantitative synthesis of the accumulated data from different studies was deemed important to provide evidence on the association between the Shenqi Jiangtang granules and type 2 diabetes. So, we carried out this meta-analysis on all published studies to estimate the therapeutic effect of Shenqi Jiangtang granules on type 2 diabetes.

## 2. Materials and Methods

### 2.1. Inclusion and Exclusion Criteria

#### 2.1.1. Type of Study

The literature of clinical RCTs related to Shenqi Jiangtang granules in the treatment of type 2 diabetes was considered, whether blind or not.

#### 2.1.2. Research Object

Patients who meet the “Chinese Type 2 Diabetes Prevention and Treatment Guidelines (2017 Version)” [18] and are diagnosed with type 2 diabetes, that is, patients with polydipsia, polyphagia, polyuria, and body mass decline, random blood glucose ≥11.1 mmol/L, fasting Blood glucose (FBG) ≥7.0 mmol/L, and/or 2 hours postprandial blood glucose (P2h BG) ≥11.1 mmol/L, were included.

#### 2.1.3. Intervention

The patients in the control group were not given treatment or simply treated with Western medicine. The patients in the test group were treated with Shenqi Jiangtang granules on the basis of the control group.

#### 2.1.4. Outcome Indicators

The main outcome indicators were effectiveness, fasting blood glucose (FBG), postprandial blood glucose (PBG), glycated hemoglobin (HbA1c), and adverse reaction rate.

#### 2.1.5. Exclusion Criteria

A large number of repeated publications; non-RCT studies (including clinical case reports, relevant literature reviews, animal pharmacological experiments, and non-RCT clinical trials and other types of literature); the literature with incomplete outcome indicators; the literature that cannot extract data; and other noncompliant inclusions standard literature were the exclusion criteria.

### 2.2. Document Retrieval Process

Relevant medical journals in Chinese and English databases, such as the China Knowledge Network (CNKI), Wanfang Data Knowledge Platform (Wanfang Data), VIP Database, and PubMed, were searched from 2014 to the present. The retrieval process follows the PICOS principle, namely, P: type 2 diabetes; I: Shenqi Jiangtang granules + Western medical method; C: western medical method; O: type 2 diabetes-related indicators; and S: randomized controlled trial. We set the following keywords as search strategies: type 2 diabetes, Shenqi Jiangtang granules, lower blood sugar, and clinical trials, and we use this search strategy to presearch Chinese and English databases, further optimize the search keywords based on the search results, and record the final search literature.

### 2.3. Data Extraction and Quality Evaluation of the Literature Methodology

After collecting and summarizing the documents retrieved above, according to the established inclusion and exclusion criteria, combined with the systematic review method of the Cochrane Collaboration Network to select qualified documents, relevant information will be extracted from the qualified documents after screening. Through the RCT quality assessment standard of the Cochrane Collaboration Network, the methodological quality of the included documents is evaluated, and the documents are included in the following 7 aspects: random method, allocation concealment, blind implementation, concealment of outcome indicators, and integrity of outcome indicators.

### 2.4. Statistical Methods

RevMan 5.2 software was used for statistical analysis of the extracted data. Binary variables and continuous variables were calculated using relative risk (RR) and mean (MD), respectively, and a 95% confidence interval (CI) was calculated. When the combined data have no significant heterogeneity (*P* > 0.10 or *I*^2^ ≤ 50%), the fixed-effect model is used for analysis; when the combined data have significant heterogeneity (*P* ≤ 0.10 or *I*^2^ ≥ 50%), then the random effects model is used for analysis. *P* < 0.05 was considered statistically significant.

### 2.5. Evidence Quality Evaluation

GraphPad Prism 6 software was used to input and quantify the quality of the included outcome indicators [19]. The GRADE evidence quality evaluation divides the outcome indicators into 3 grades, of which 1–3 are unimportant outcome indicators, 4–6 are important outcome indicators, and 7–9 are key outcome indicators; RCT is set as the high quality of intervention effect estimation evidence, and observational studies were set as low-quality evidence. Based on the research design, it can be divided into 5 downgrade factors and 4 upgrade factors [20]. The main research object of this article is RCT articles, mainly based on the risk of bias, inaccuracy, inconsistency, indirectness, and publication bias to classify the quality of evidence.

## 3. Results

### 3.1. Literature Screening Process and Results

**Figure 1:**
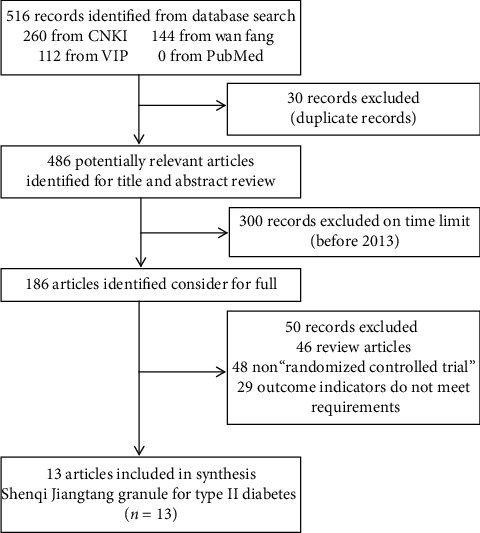
Document screening flow chart.

A total of 516 related documents were retrieved through the system. After screening duplicate documents, limiting publication time, and reading titles and abstracts, a total of 503 articles were excluded, and finally, 13 articles were included. The specific screening process is shown in Figure 1.

### 3.2. Basic Information Included in the Literature

All studies are conducted in China and are RCT experiments.  Table 1 shows the detailed characteristics of all studies, such as age, sex ratio, intervention methods, treatment cycle, and outcome indicators. A total of 1160 patients were tested in this study, including 580 in the test group (T) and 580 in the control group (C).

### 3.3. Inclusion of Literature Quality Evaluation

**Figure 2:**
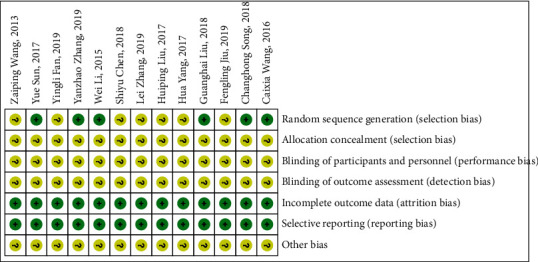
Document quality evaluation chart.

Refer to the bias risk assessment standard provided by the Cochrane Collaboration Network to conduct randomization, allocation concealment, blind implementation, blindness of outcome indicators, completeness of outcome indicators, and other 7 aspects of bias studies for the included documents. It is expressed in three ways: low risk, high risk, and unclear. The results are shown in Figure 2.

### 3.4. Meta-Analysis Results

#### 3.4.1. Meta-Analysis Based on the Effectiveness of Clinical Treatment

**Figure 3:**
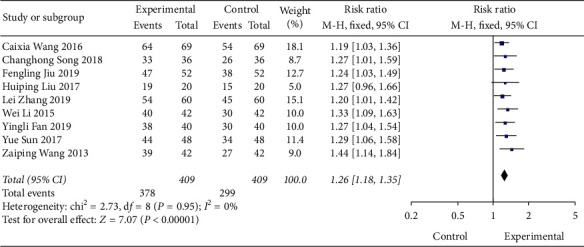
Forest map of the effective rate of Shenqi Jiangtang granule in the treatment of type 2 diabetes mellitus.

**Figure 4:**
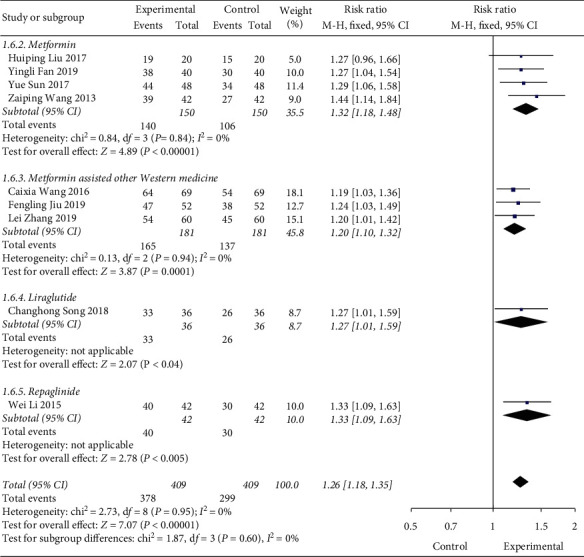
Forest map of subgroup analysis on the effective rate of Shenqi Jiangtang granule in the treatment of type 2 diabetes mellitus.

Among the 13 studies included, 9 articles included statistics on the total effectiveness of the experimental group and the control group, and the analysis showed no heterogeneity (DF = 8, *P*=0.95, *I*^2^ = 0%), so a fixed-effect model is used. Meta-analysis results are shown in Figure 3. The combined RR value of the included literature is 1.26 (95% CI (1.18, 1.34)). As shown in Figure 4, we performed a subgroup analysis of the included studies according to the type of treatment drug in the control group. The results showed that there was no heterogeneity in each subgroup. The results show that the total effective rate of the experimental group is higher than that of the control group, and the difference is statistically significant (*P* < 0.00001).

#### 3.4.2. Meta-Analysis Based on Fasting Blood Glucose Indicators

**Figure 5:**
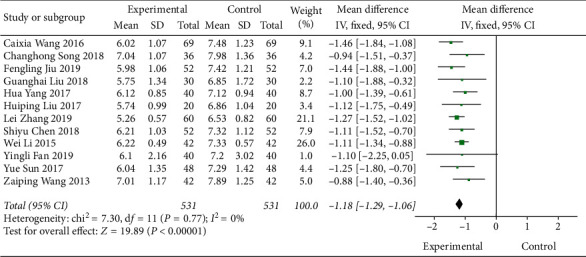
Forest map of FBG of Shenqi Jiangtang granule in the treatment of type 2 diabetes mellitus.

**Figure 6:**
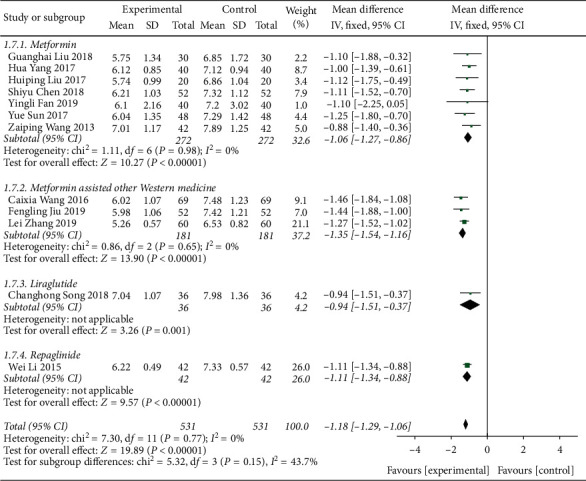
Forest map of subgroup analysis on the FBG of Shenqi Jiangtang granule in the treatment of type 2 diabetes mellitus

A total of 12 literature were included in the fasting blood glucose test. The results of the study showed that there was no heterogeneity between the studies (DF = 11, *P*=0.77, *I*^2^ = 0%). The fixed-effect model was used.  Figure 5 shows that the fasting blood glucose control degree of the Shenqi Jiangtang granules combined medication group was better than that of the control group, MD = −1.18 (95% CI (−1.29, −1.06)), and the results of the test group and the control group were statistically different (*P* < 0.00001). As shown in Figure 6, we conducted a subgroup analysis of the included studies based on the type of therapeutic drugs in the control group. The results showed that there was no heterogeneity in each subgroup. The results showed that the fasting blood glucose index of the experimental group was lower than that of the control group, and the difference was statistically significant.

#### 3.4.3. Meta-Analysis Based on Postprandial Blood Glucose Indicators

**Figure 7:**
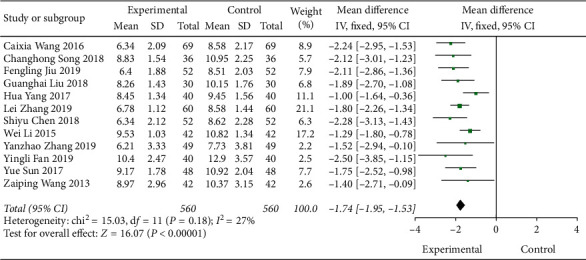
Forest map of PBG of Shenqi Jiangtang granule in the treatment of type 2 diabetes mellitus.

**Figure 8:**
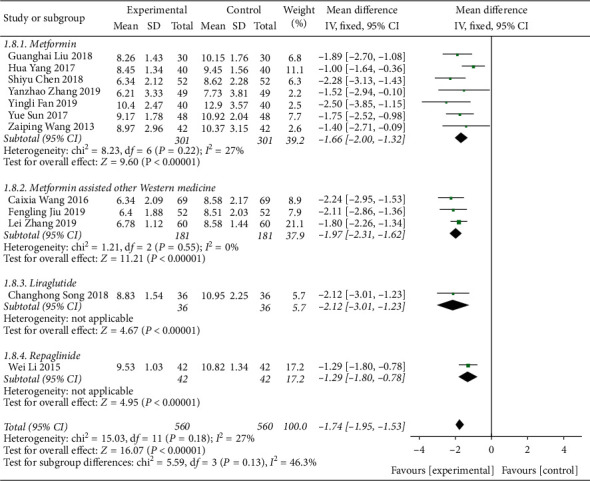
Forest map of subgroup analysis on the PBG of Shenqi Jiangtang granule in the treatment of type 2 diabetes mellitus.

A total of 12 literature were included in the postprandial blood glucose test, and the results showed that there was no heterogeneity (DF = 11, *P*=0.18, *I*^2^ = 27%); a fixed-effect model was used.  Figure 7 shows that Shenqi Jiangtang granules degree of postprandial blood glucose control in the combined medication group was better than that in the control group, MD = −1.74 (95% CI (−1.95, −1.53)). The results of the test group and the control group were statistically different (*P* < 0.00001) (see Figure 7). As shown in Figure 8, we conducted a subgroup analysis of the included studies based on the type of therapeutic drugs in the control group. The results showed that there was no heterogeneity in each subgroup. The results showed that the postprandial blood glucose index of the experimental group was lower than that of the control group, and the difference was statistically significant.

#### 3.4.4. Meta-Analysis Based on the Glycated Hemoglobin Index

**Figure 9:**
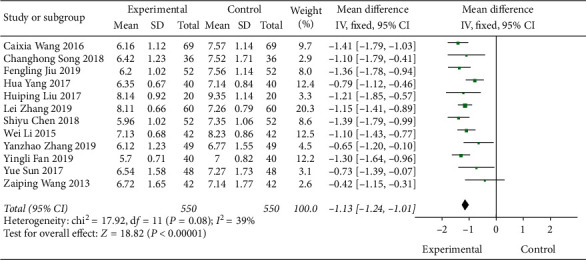
Forest map of HbA1c of Shenqi Jiangtang granule in the treatment of type 2 diabetes mellitus.

**Figure 10:**
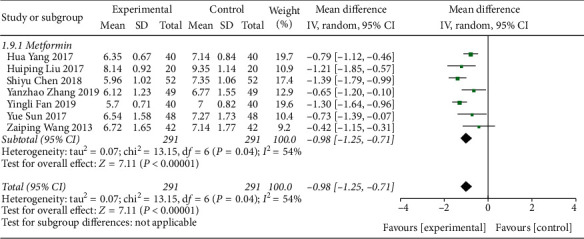
Forest map of subgroup analysis on the HbA1c of Shenqi Jiangtang granule in the treatment of type 2 diabetes mellitus.

**Figure 11:**
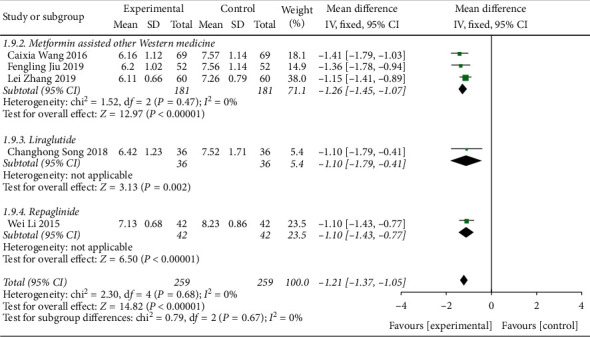
Forest map of subgroup analysis on the HbA1c of Shenqi Jiangtang granule in the treatment of type 2 diabetes mellitus.

A total of 12 literature were included in the detection of outcome indicators for glycated hemoglobin. The results of the study showed that there was no heterogeneity between the studies (DF = 11, *P*=0.08, *I*^2^ = 39%). A fixed-effect model was used.  Figure 9 shows that the control level of glycated hemoglobin in the Shenqi Jiangtang granules combined medication group was better than that in the control group, MD = −1.13 (95% CI (−1.24, −1.01)), and the results of the experimental group and the control group were statistically different (*P* < 0.00001). As shown in Figures 10 and 11, we conducted a subgroup analysis of the included studies based on the type of therapeutic drugs in the control group. The results showed that the metformin control group was heterogeneous, so a random effect model was used. The heterogeneity effect is small and may be caused by clinical heterogeneity. There was no heterogeneity in the remaining subgroups. The results showed that the glycated hemoglobin index of the experimental group was lower than that of the control group, and the difference was statistically significant.

#### 3.4.5. Meta-Analysis of Adverse Reactions

**Figure 12:**
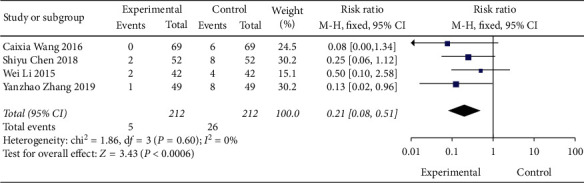
Forest map of subgroup analysis of adverse reactions of Shenqi Jiangtang granule in the treatment of type 2 diabetes mellitus.

**Figure 13:**
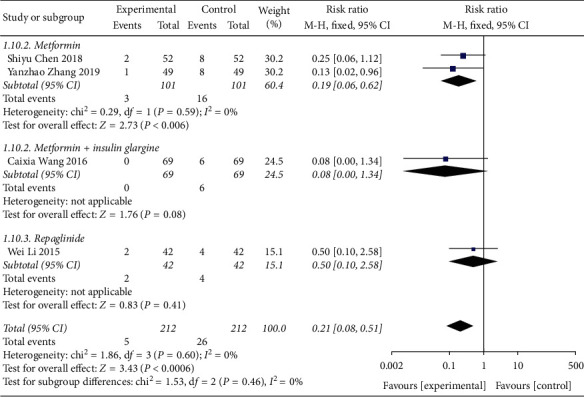
Forest map of subgroup analysis of adverse reactions of Shenqi Jiangtang granule in the treatment of type 2 diabetes mellitus.

Adverse events were mentioned in the 4 articles included, and the results showed no heterogeneity between studies (DF = 3, *P*=0.6, *I*^2^ = 0%), using a fixed-effect model.  Figure 12 shows the combination of Shenqi Jiangtang granules The incidence of adverse reactions in the treatment group was lower than that in the control group MD = 0.21 (95% CI (0.08, 0.51)), and there was a significant difference between the experimental group and the control group (*P*=0.0006). As shown in Figure 13, we conducted a subgroup analysis of the included studies based on the type of therapeutic drugs in the control group. The results showed that there was no heterogeneity in each subgroup. The results showed that the adverse reactions of the experimental group were lower than that of the control group, and the difference was statistically significant.

#### 3.4.6. Sensitivity Analysis

Sensitivity analysis of the efficacy, fasting blood glucose, postprandial blood glucose, glycated hemoglobin, and adverse reaction rate of Shenqi Jiangtang granules in the treatment of type 2 diabetes was performed by changing the effect model and removing the larger or smaller proportion of the weights. The results of the study did not change significantly, indicating that our results were statistically reliable, as shown in Table 2.

#### 3.4.7. Evaluation of Publication Bias Based on the Total Effective Clinical Efficacy

**Figure 14:**
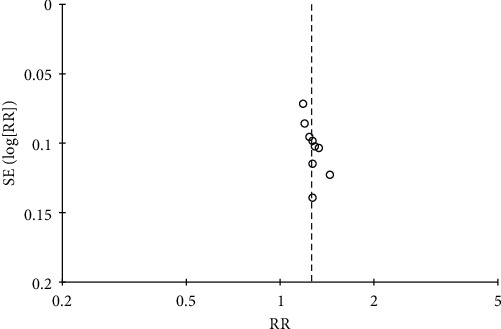
Funnel plot of the total efficacy of SQJT granules compared with conventional medicines.

Evaluation of the total effectiveness of the included literature is performed, as shown in Figure 14, and the funnel chart results suggest that the study data of Shenqi Jiangtang granules combined with conventional Western medicine in the treatment of type 2 diabetes are more authentic and less likely to be biased.

### 3.5. Evaluation of the Evidence Quality

**Figure 15:**
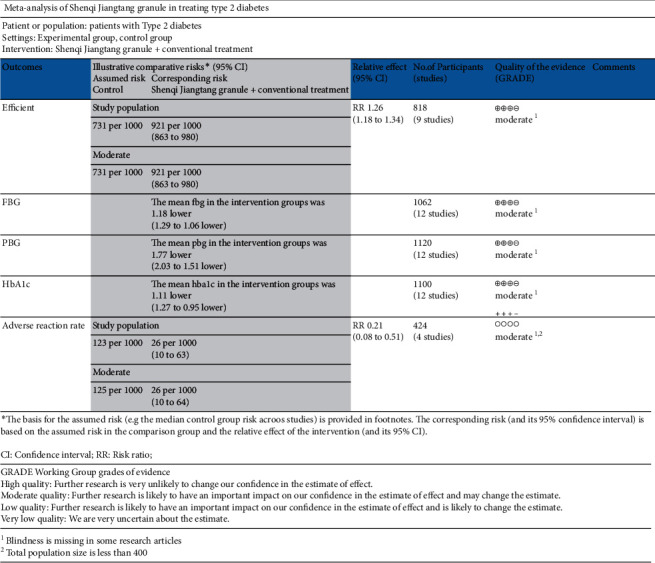
Grading evaluation chart of evidence quality.

GraphPad Prism 6 was used to evaluate the quality of the included literature. The total effective rate of treatment is a key outcome indicator, and fasting blood glucose, postprandial blood glucose, and glycated hemoglobin are important outcome indicators. The grading chart of evidence quality of each outcome index is shown in Figure 15. All included documents are RCT experiments, and there is no obvious publication bias.

## 4. Discussion

As a high-risk group, type 2 diabetes patients are increasingly valued by more and more medical workers, which has also caused a lot of attention. The patient's body has been in a disorder of blood glucose and blood lipid metabolism for a long time, and the possibility of various chronic complications has also increased significantly [34]. Western medicine hypoglycemic drugs achieve a good hypoglycemic effect by enhancing the body's consumption of glucose, inhibiting its absorption and production [35]. However, in the long-term observation of Western medicine treatment, it has been found that insulin secreted and synthesized by islet *β* cells under physiological conditions has no obvious hypoglycemic effect, the feedback of insulin concentration increases, and the patient's blood glucose level increases significantly. Long-term resistance of the islet function not only affects the hypoglycemic effect of the drug but also causes certain damage to islet *β* cells, resulting in functional exhaustion [36]. “Su Wen·Singular Disease” records that “this person must eat sweet and fat but also fat so his qi overflowed and turned to thirst.” Obesity is the basis for the onset of type 2 diabetes, and phlegm turbidity, dampness, and heat content are the initial factors. Phlegm turbidity, dampness, and heat obstruction are scorching, soil stagnation, spleen loss of health, liver loss and drainage, and water valley fineness. Stagnation of blood is an important part of the rise of blood sugar and its incidence, and blood stasis is the main cause of various comorbidities [37]. The main components of Shenqi Jiangtang granules are ginseng, ginsenosides, *Astragalus*, *Ophiopogon japonicus*, raspberry, trichosanthin, *Rehmannia glutinosa*, poria, medlar, *Alisma*, *Schisandra*, and yam. Modern pharmacological studies have shown that ginsenoside can repair islet *β* cells, promote insulin release, and can inhibit alloxan and improve hyperglycemia [38]. *Astragalus* can regulate the body's immunity, scavenge oxygen free radicals, protect vascular endothelial cells, increase insulin sensitivity, weaken insulin resistance, and regulate blood lipid and blood glucose. Poria can lower blood sugar and increase the function of islet *β* cells. Studies have shown that the use of poria can increase the antioxidant capacity of kidneys in type 2 diabetic mice and plays a protective role in the kidneys. Ginseng can also increase the sensitivity of insulin and effectively regulate blood sugar and blood lipids; in addition, it can increase the antioxidant capacity of the myocardium, protect vascular endothelial cells, and inhibit cardiomyocyte apoptosis [39]. *Ophiopogon japonicus* has a strong lipid-lowering effect and can reduce the content of TC in blood [40]. *Schisandra* can inhibit glucosidase and plays a role in lowering blood sugar [41]. The combined use of various drugs can play a role in regulating the body and has obvious therapeutic effects on stress hyperglycemia, lipid peroxide after abnormal glucose metabolism, and insulin-damaging hyperglycemia [42].

In this study, after the intervention of the experimental group through Shenqi Jiangtang granules combined with conventional Western medicine treatment and the control group with only conventional Western medicine treatment, the main outcome indicators showed a treatment trend, but the improvement of the indicators of the patients in the experimental group was significantly greater than that of the control group. For the patients in the group, the difference in data was statistically significant (*P* < 0.05). First, it shows the therapeutic effect of Western medicine conventional hypoglycemic drugs on type 2 diabetes and also suggests that Shenqi Jiangtang granules can significantly improve the effectiveness of clinical treatment based on Western medicine treatment and further improve PBG, FBG, HbA1c, and other related indicators.

Limitations in our meta-analysis should be considered as follows: First of all, the literature was included with low quality and small sample sizes. Also, the research methods were not reported in details, thereby making bias risk assessment difficult. Particularly, none of the studies provided any detail on single or double blinding and allocation concealment, which indicated poor quality of the methodology and led to high risk of selection and measurement bias. Secondly, although we adopted an adequate search strategy to minimize publication bias, some potential biases may still exist because of language restriction. Thirdly, drug safety is a key factor in clinical applications, but only four RCTs described adverse reactions or events. Therefore, the safety of using Shengqi Jiangtang granule should be validated in future to bring more convincing evidence. Besides, none of the studies reported end-point outcomes such as the incidence of type 2 diabetes, fatality rate, and life quality, thus making the assessment of the long-term efficacy of Shenqi Jiangtang granule difficult, which will affect the further development of drugs.

Findings from this meta-analysis illustrate that Shenqi Jiangtang granule in the treatment of type 2 diabetes mellitus may be effective. Because of the poor methodological quality and small sample sizes, further validation is essential. Therefore, we recommend the conduction of multicenter, large-sample, and randomized controlled double-blind trials to provide more accurate and reliable evidence for clinical research.

## Figures and Tables

**Table 1:** Principal characteristics of the studies included in the meta-analysis.

Reference	Sample size (C/T)	Gender (male/female)		Age (range, mean)		Treatment method		Treatment cycle (week)	Measurement index
Zhang et al., 2019 [21]	60/60	T: 31/29	C: 33/27	T: 45–67 (57.13 ± 9.86)	C: 42–69 (57.46 ± 9.74)	T: SQJT + Metformin + Glimepiride	C: Metformin + Glimepiride	12	1、2、3、4
Sui 2019 [22]	52/52	T: 30/22	C: 31/21	T: 39–68 (54.31 ± 5.11)	C: 38–67 (53.25 ± 4.70)	T: SQJT + Metformin + Rosiglitazone	C: Metformin + Rosiglitazone	12	1、2、4
Liu and Gao, 2017 [23]	20/20	T: 23/14	C: 10/10	T: 41–65 (56.55 ± 7.17)	C: 42–65 (55.30 ± 7.36)	T: SQJT + Metformin	C: Metformin	12	1、2、3、4
Sun, 2017 [24]	48/48	T: 28/20	C: 30/18	T: 38–64 (56.27 ± 4.61)	C: 36–62 (54.15 ± 4.29)	T: SQJT + Metformin	C: Metformin	8	1、2、3、4
Wang, 2016 [25]	69/69	T: 43/26	C: 41/28	T: 25–65 (54.3 ± 3.2)	C: 26–65 (53.2 ± 3.4)	T: SQJT + Metformin + Insulin glargine	C: Metformin + Insulin glargine	12	1、2、3、4
Yang, 2017 [26]	40/40	T: 21/19	C: 22/18	T: 42–65 (55.8 ± 8.9)	C: 45–64 (55.4 ± 6.8)	T: SQJT + Metformin	C: Metformin	24	2、3、4
Chen, 2018 [27]	52/52	T: 32/20	C: 31/21	T: 26–63 (55.2 ± 3.5)	C: 27–60 (54.3 ± 3.7)	T: SQJT + Metformin	C: Metformin	12	2、3、4
Fan and Gao, 2014 [28]	40/40	T: 22/18	C: 24/16	T: 42–74	C: 40–73	T: SQJT + Metformin	C: Metformin	12	1、2、3、4
Zhang, 2019 [29]	49/49	T: 29/20	C: 31/18	T: 40–75 (59.18 ± 7.66)	C: 43–74 (60.27 ± 6.34)	T: SQJT + Metformin	C: Metformin	12	3、4
Song, 2018 [30]	36/36	T: 21/15	C: 20/16	T: 43–76 (59.12 ± 10.83)	C: 41–74 (58.94 ± 10.15)	T: SQJT + Liraglutide	C: Liraglutide	12	1、2、3、4
Li, 2015 [31]	42/42	T: 27/15	C: 26/16	T: 53–68 (63.1 ± 6.6)	C: 54–69 (62.8 ± 6.2)	T: SQJT + Repaglinide	C: Repaglinide	4	1、2、3、4
Wang, 2013 [32]	42/42	T: 26/16	C: 27/15	T: 35–66 (45.9 ± 7.4)	C: 33–63 (43.5 ± 5.2)	T: SQJT + Metformin	C: Metformin	8	1、2、3、4
Liu, 2018 [33]	30/30	T: 14/16	C: 13/17	T: 60–76 (67.2 ± 9.4)	C: 60–78 (66.8 ± 10.2)	T: SQJT + Metformin	C: Metformin	12	2、3

*Note.* 1. Efficient; 2. FBG; 3. PBG; 4. HbA1c.

**Table 2:** Sensitivity analysis of Shenqi Jiangtang granule in treating type 2 diabetes.

Outcome indicators	Fixed effect model			Random effect model			Remove big weight			Remove small weight		
	MD	95% CI	*P*	MD	95% CI	*P*	MD	95% CI	*P*	MD	95% CI	*P*
Efficient	1.26	[1.18, 1.35]	0.0001	1.26	1.18–1.34	0.0001	1.28	1.19–1.38	0.0001	1.26	1.18–1.35	0.0001
FBG	−1.18	[−1.29, −1.06]	0.0001	−1.18	[−1.29, −1.06]	0.0001	−1.2	[−1.33, −1.07]	0.0001	−1.18	[−1.29, −1.06]	0.0001
PBG	−1.74	[−1.95, −1.53]	0.0001	−1.77	[−2.03, −1.51]	0.0001	−1.72	[−1.96, −1.48]	0.0001	−1.74	[−1.96, −1.53]	0.0001
HbA1c	−1.13	[−1.24, −1.01]	0.0001	−1.11	[−1.27, −0.95]	0.0001	−1.1	[−1.28, −0.91]	0.0001	−1.14	[−1.29, −0.99]	0.0001
Adverse reaction	0.21	[0.08, 0.51]	0.0001	0.24	[0.1, 0.6]	0.0001	0.24	[0.06, 0.92]	0.0001	0.16	[0.05, 0.47]	0.001

## Data Availability

The data used to support the findings of this study are included within the article.
